# The Application Value of Metagenomic and Whole-Genome Capture Next-Generation Sequencing in the Diagnosis and Epidemiological Analysis of Psittacosis

**DOI:** 10.3389/fcimb.2022.872899

**Published:** 2022-06-06

**Authors:** Zhimei Duan, Yanqiu Gao, Bin Liu, Baohua Sun, Shuangfeng Li, Chenlei Wang, Dongli Liu, Kaifei Wang, Ye Zhang, Zheng Lou, Lixin Xie, Fei Xie

**Affiliations:** ^1^ College of Pulmonary and Critical Care Medicine, Chinese People’s Liberation Army (PLA) General Hospital, Beijing, China; ^2^ Respiratory Intensive Care Unit, Zhengzhou Central Hospital Affiliated to Zhengzhou University, Zhengzhou, China; ^3^ Characteristic Medical Center, the Chinese People’s Armed Police Forces, Tianjin, China; ^4^ Department of the Respiratory and Critical Care Medicine, Cangzhou Central Hospital, Cangzhou, China; ^5^ Department of the Respiratory and Critical Care Medicine, Yan’an University Affiliated Hospital, Yan’an, China; ^6^ Department of Scientific Affairs, Hugobiotech Co., Ltd., Beijing, China

**Keywords:** Psittacosis, *Chlamydophila psittaci*, metagenomic next-generation sequencing, capture, diagnosis, epidemiology

## Abstract

**Background:**

To evaluate the value of metagenomic next-generation sequencing (mNGS) for the early diagnosis of psittacosis, and to investigate its epidemiology by whole-genome capture.

**Methods:**

Twenty-one bronchoalveolar lavage fluid (BALF) and blood samples of 16 psittacosis patients from multiple centers during August 2019 to September 2021 were analyzed retrospectively. mNGS with normal datasets (10 M 75-bp single-end reads after sequencing) and larger datasets (30 M 150-bp paired-end reads after sequencing) as well as quantitative real-time polymerase chain reaction (qPCR) were used to detect the pathogen. Also, whole-genome capture of *Chlamydophila psittaci* was applied to draw the phylogenetic tree.

**Results:**

mNGS successfully detected the pathogen in all 16 cases (100%), while qPCR was positive only in 5 out of 10 cases (50%), indicating a significantly higher sensitivity of mNGS than qPCR (*p* < 0.01). BALF-mNGS performed better than blood-mNGS (16/16 versus 3/5, *p* < 0.05). In addition, larger datasets (the read counts have tripled, and the base number was 12-fold larger compared to clinical mNGS with a normal dataset) of mNGS showed significantly increased contents of human DNA (*p* < 0.05) and decreased reads per million of the pathogen, suggesting no improvement. Whole-genome capture results of five samples (>60% coverage and >1 depth) were used to construct the phylogenetic tree.

**Conclusion:**

Significant advantages of mNGS with normal datasets were demonstrated in early diagnosing psittacosis. It is the first study to use whole-genome capture to analyze *C. psittaci* epidemiological information.

## Introduction

**Figure 1:**
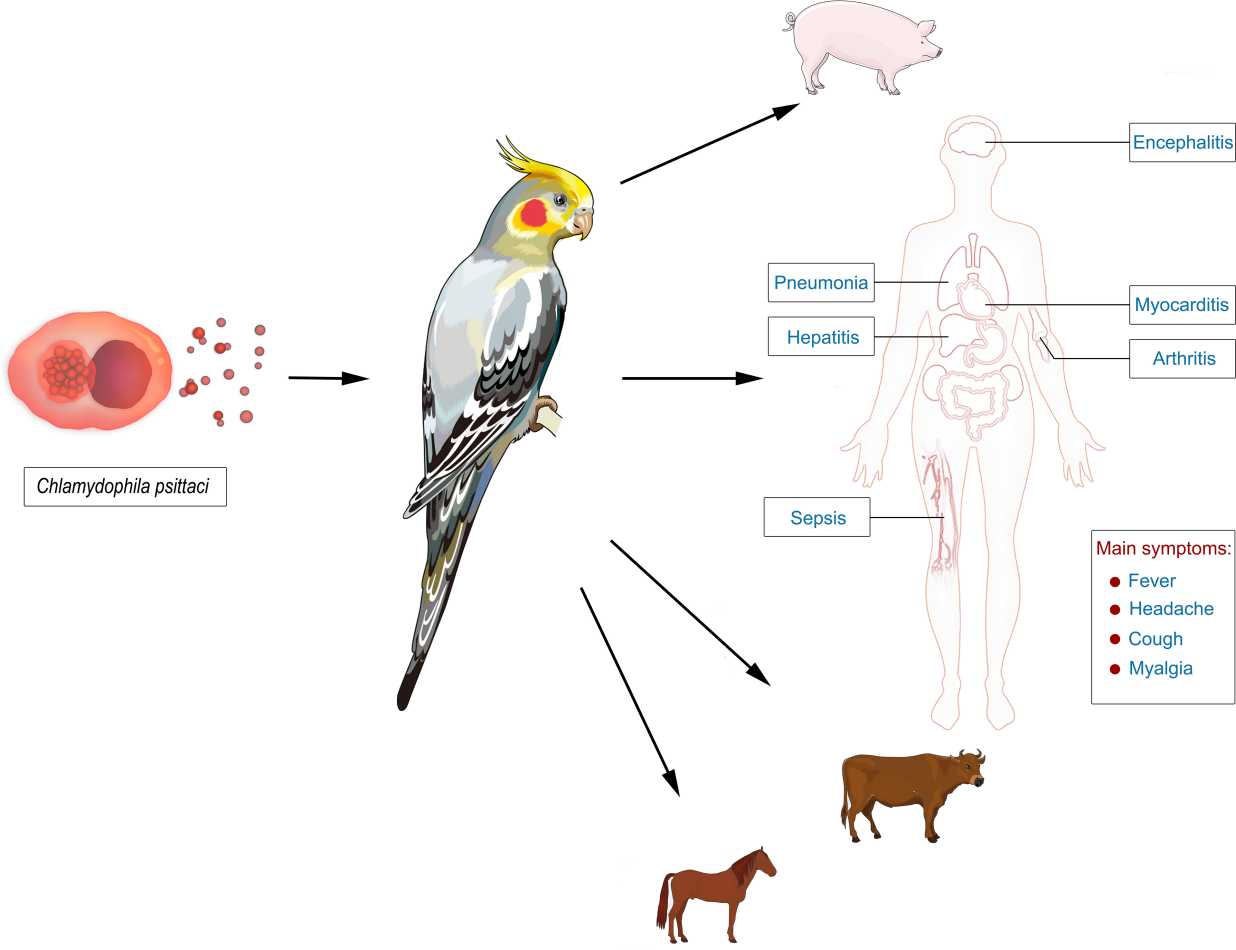
The epidemiology of psittacosis and its manifestations in infected persons. Psittacosis is a zoonotic disease, and birds are the major source of infection. The pathogen, *Chlamydophila psittaci*, can infect multiple organs of the body and cause pneumonia, septic shock, and multiple-organ failure. The typical clinical symptoms include fever, headache, cough, and myalgia.

Psittacosis, also called ornithosis or parrot fever, is a zoonotic disease caused by *Chlamydophila psittaci* (Stewardson and Grayson, 2010). Birds are the major source of infection (Kaleta and Taday, 2003). Most patients with psittacosis have a history of contact with birds, including parrots, pigeons, ducks, turkeys, and chickens (Kaleta and Taday, 2003; Stewardson and Grayson, 2010). Human-to-human transmission is rare (Ito et al., 2002). Psittacosis has a worldwide distribution and can infect humans with a low incidence (~1% in community-acquired pneumonia) (Speer, 2016). For example, the number of reported cases of psittacosis in the United States during 2008~2017 was 60 (Shaw et al., 2018 2019), whereas only 115 psittacosis cases were found from 2007 to 2016 in Japan (Kozuki et al., 2020). However, it is thought that psittacosis occurs more frequently than reported given conventional clinical tests of *C. psittaci* are not available in most hospitals (Gregory and Schaffner, 1997). In addition, not all infections lead to pneumonia and therefore often remain unnoticed and are not considered until the patients have no response to β-lactam antibiotic treatments (Gregory and Schaffner, 1997; Rybarczyk et al., 2020). It is worth noting that multiple psittacosis outbreaks have been reported worldwide (Beeckman and Vanrompay, 2009; Brass, 2016; Shaw et al., 2018 2019; Kozuki et al., 2020). Psittacosis can cause flu-like symptoms, with an incubation period of 1~4 weeks (Beeckman and Vanrompay, 2009). The predominant syndrome is respiratory tract infection (Crosse, 1990), with typical clinical symptoms including fever, headache, cough, and myalgia (Yung and Grayson, 1988; Moroney et al., 1998), while other clinical symptoms may include atypical pneumonia, septic shock, and multiple-organ failure (**Figure 1**) (Stewardson and Grayson, 2010). The severity and clinical features of psittacosis range widely among patients (Crosse, 1990; Heddema et al., 2006). Most patients respond to proper antibiotic treatments and experience a good prognosis. However, severe features involving multiple systems of the body may occur, even resulting in death (Byrom et al., 1979). The mortality rate is 1% (Brass, 2016). The early diagnosis of this disease can help the timely adjustment of antibiotic treatments, significantly improving the prognosis of patients.

The clinical diagnosis of psittacosis remains difficult. Similar symptoms to other flu-like diseases and variation in different patients (Stewardson and Grayson, 2010) result in many misdiagnosed cases. In addition, conventional diagnostic methods, including culture, serology, and polymerase chain reaction (PCR), are not ideal for the diagnosis of psittacosis (Smith et al., 2005; Branley et al., 2008). Most laboratories do not have the capability of performing *C. psittaci* culture, which must be cultivated in tissue culture, mice, or chick embryo, taking several weeks (William et al., 2000). Serology is the most commonly used method in many clinical laboratories, but the sensitivity and specificity are low, especially for the early diagnosis (Smith et al., 2005). PCR is fast with a relatively high sensitivity and specificity, but it needs a prior hypothesis of the target (Mitchell et al., 2009), limiting its application in clinical diagnoses. By contrast, the state-of-the-art metagenomic next-generation sequencing (mNGS) has been successfully applied in diagnosing multiple clinical diseases, including sepsis, meningitis, and pneumonia (Chiu and Miller, 2019; Chen et al., 2021). It is culture-independent and able to rapidly detect almost all pathogens, including bacteria, viruses, fungi, parasites, and chlamydia (Gu et al., 2021). Compared to the conventional methods, mNGS is less affected by previous antibiotic treatment, and the sensitivity and specificity are always higher, suggesting its great advantages, especially for rare, novel, and atypical infectious diseases (Gu et al., 2021). Notably, the sensitivity of mNGS and qPCR always depends on the load of pathogens in samples. A low pathogen load can lead to missed diagnosis by either diagnostic method. Theoretically, we can get more pathogen reads by sequencing for higher throughput but may also bring more DNA reads of human host and background microorganisms. The diagnosis value of mNGS with normal and enlarged datasets has been less discussed.

In this study, patients with psittacosis were enrolled for a multicenter retrospective analysis, with the aim to evaluate the value of mNGS in diagnosing psittacosis compared to quantitative real-time PCR (qPCR) using blood and bronchoalveolar lavage fluid (BALF) samples. We also analyzed the impact of applying larger datasets of mNGS to the diagnosis. In addition, whole-genome capture of *C. psittaci* was also applied in this study. A phylogenetic tree was built to illustrate its epidemiological spectrum.

## Material and Methods

### Sample Collection

Sixteen patients hospitalized and diagnosed with psittacosis, from August 2019 to September 2021, were enrolled for this multicenter retrospective study. These patients were from five hospitals, including Chinese PLA General Hospital, Zhengzhou Central Hospital Affiliated to Zhengzhou University, the Chinese People’s Armed Police Forces, Cangzhou Central Hospital, and Yan’an University Affiliated Hospital. Their baseline information and clinical data were recorded. BALF and blood samples were also collected.

All procedures performed in this study involving human participants were in accordance with the ethical standards of the institutional and/or national research committee(s) and with the Helsinki Declaration (as revised in 2013). This study was approved by the ethical review committee of General Hospital of the People’s Liberation Army (No. S2019-266-02). Written informed consent was obtained from the patients.

### Molecular Detection of Samples

A total of 21 samples were collected from the 16 patients, including 16 BALF and 5 blood samples, and were transferred for PACEseq mNGS (Hugobiotech, Beijing). DNA was extracted using QIAamp DNA Micro Kit (QIAGEN, Hilden). DNA libraries were then built using QIAseq™ Ultralow Input Library Kit (QIAGEN, Hilden) according to its manufacturer’s instructions. Qubit (Thermo Fisher, Waltham, MA) and Agilent 2100 Bioanalyzer (Agilent Technologies, Palo Alto) were applied to evaluate the quality of libraries. For clinical mNGS detection, all qualified libraries were sequenced on a NextSeq 550Dx platform (Illumina, San Diego) with a normal dataset (~10 M 75-bp single-end reads after sequencing). For mNGS detection with a larger dataset, paired-end sequencing using the same libraries was performed on a NovaSeq 6000 platform (Illumina, San Diego), and ~30 M 150-bp paired-end reads were obtained. The larger dataset resulted in the read count tripling and the base number being 12-fold larger compared to the normal clinical mNGS dataset.

Short reads, low-quality reads, and low complexity reads were removed from the raw data. Human host DNA were then filtered out after aligning to the human reference database (hg38). The clean reads were aligned to Microbial Genome Databases (http://ftp.ncbi.nlm.nih.gov/genomes/) using BWA. The read number and reads per million (RPM) of each detected pathogen were calculated. For detected microbes, including bacteria (*Mycobacteria* excluded), fungi (*Cryptococcus* excluded), and parasites, a positive mNGS result was given when its coverage ranked in the top 10 of similar microbial species (or genera) and was absent in the negative control (“No template” control, NTC) or when its ratio of RPM between sample and NTC (RPM_sample_/RPM_NTC_) > 10 if RPM_NTC_ ≠ 0. For viruses, *M. tuberculosis*, *C. psittaci*, and *Cryptococcus*, a positive mNGS result was considered when at least one unique read was mapped to species level and absent in NTC or RPM_sample_/RPM_NTC_ > 5 when RPM_NTC_ ≠ 0.

Whole-genome capture of *C. psittaci* was also applied in this study. The probes were designed in 200 bp of length. The detailed information of the probes, including the sequences and co-ordinates of the target, was shown in the supplementary materials. The whole genome of *C. psittaci* of each sample was then captured using a Twist-customized panel (Twist, San Francisco) based on Twist Fast Hybridization Target Enrichment Protocol in this study. The enriched libraries were then sequenced on NovaSeq 6000 (Illumina, San Diego). After removing the short or low-quality reads and human host reads, the clean data were aligned to the *C. psittaci* reference database (GCF_000204255.1). The coverage and mean depth of each sample were calculated.

qPCR was also used to detect the pathogen, and a 172-bp DNA-specific fragment on the *ompA* gene was detected. The primers were AGAAGCAAATGGCAGACCGA (F) and AACCAAGTTGAATGCAGCCGA (R). Samples with a Ct value of qPCR < 37 were considered as positive. Clone and Sanger sequencing was performed to verify the qPCR detection.

### Data Analysis

The sensitivity and positive rate of mNGS and qPCR against the final diagnosis were calculated in this study. The value of mNGS in diagnosing psittacosis was evaluated by comparing to that of qPCR. To better understand if larger datasets of mNGS can help the diagnosis, the detection results of mNGS with normal datasets and larger datasets (the read counts have tripled, and the base number was 12-fold larger compared to clinical mNGS with normal dataset) were compared. The mNGS results detected by different types of samples (BALF and blood) were also compared. R software (version 4.1.0) was applied for the statistical analysis, including Fisher test and Wilcoxon’s test using the Stats package.

The coverage and mean depth of *C. psittaci* after whole-genome capture were calculated. The genetic results with >60% coverage and >1 mean depth were then used to build the phylogenetic tree by MEGA X based on the maximum-likelihood method and neighbor-joining method (bootstrap = 1,000). The genetic relationship of different *C. psittaci* strains worldwide was analyzed. The relationship of each paired *C. psittaci* strains of this study was analyzed by comparing the differences of variations in all shared single-nucleotide polymorphisms (SNPs). We also processed and assembled the reads of samples with high-quality capture results (coverage >99% and mean depth >100) using megahit. The nucleotide identity of different strains was calculated using fastANI. The amino acid sequence of cases with coverage >99% and mean depth >100 was predicted by Prokka.

## Results

### The Baseline of Patients

A total of 16 patients were enrolled in this study, including seven from the General Hospital of the People’s Liberation Army, four from Zhengzhou Central Hospital, two from Military Medical Center, two from Cangzhou Central Hospital, and one from Yan’an University Affiliated Hospital.

Nine out of the 16 (56%) patients were men, and seven (44%) were women. The average age was 57 (16~88) years. Ten patients (63%) had underlying diseases, including diabetes, hypertension, coronary heart disease, and liver cirrhosis. Nine patients (56%) had a history of exposure to birds, such as parrots, pigeons, or poultry. Their main symptoms included fever and cough, accompanied by chills, fatigue, chest tightness, and diarrhea, all of which were common for psittacosis. The involved systems included respiratory system, digestive system, urinary system, blood system, circulatory system, and nervous system. Pneumonia was the most common illness of these patients. CT results of the patients revealed consolidation, nodules, and ground-glass shadows distributed along the subpleura, accompanied by a small amount of pleural effusion. Blood routine tests revealed 14 patients (87.5%) with a normal range (3.5~10 ×10^9^/l) of white blood count (WBC), consistent with previous studies (Branley et al., 2014). The detailed baseline of patients is shown in **Table S1**. All patients received 2–4 weeks of tetracyclines and/or new fluoroquinolone therapy. They were finally cured with good prognosis.

### The Comparison Between mNGS and qPCR

In this study, the value of mNGS and qPCR in diagnosing psittacosis was compared. mNGS was performed in all 21 samples (16 BALF and 5 blood samples) from the 16 cases. mNGS detected *C. psittaci* in all cases; the sensitivity of mNGS was 100%. Except two blood samples, all samples showed positive *C. psittaci*; the detection rate of mNGS was 90.48%.

**Figure 2:**
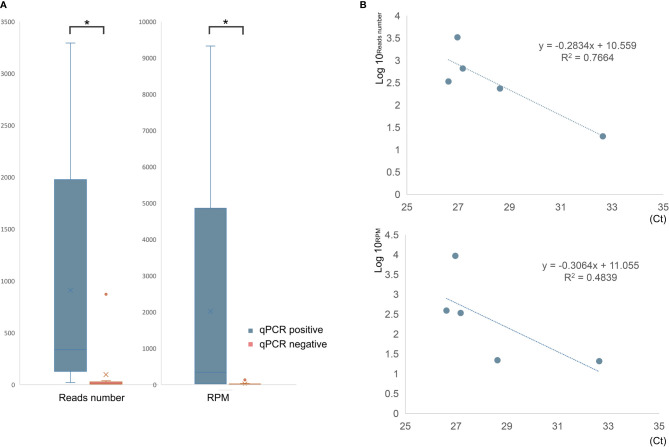
The comparison of mNGS results between qPCR-positive and -negative samples. **(A)** The read number and RPM of *C. psittaci* were significantly higher in qPCR-positive samples than that of negative samples. **(B)** The relationship between reads number (top) or RPM (bottom) and cycle threshold (Ct) value of qPCR. *p < 0.05.

qPCR and mNGS were performed simultaneously in 15 samples (10 BALF and 5 blood samples) of 10 patients. Only five BALF samples were detected with a confirmed targeted *C. psittaci* fragment by qPCR. The sensitivity and detection rate of qPCR were 50% and 33%, respectively. By contrast, mNGS successfully detected the pathogen in all the 10 cases, and only two blood samples were negative. The sensitivity and detection rate of mNGS in these patients were 100% and 87%, respectively. The detection rate and sensitivity of mNGS were significantly higher than those of qPCR (*p <*0.01). In addition, the difference in mNGS pathogens’ specific read number and RPM between samples with positive and negative qPCR results were calculated and compared. It was found that the specific read number and RPM of *C. psittaci* in samples with positive qPCR results were significantly higher than those that failed to detect the pathogen by qPCR (*p* < 0.05) (**Figure 2**). In other words, mNGS detected the pathogen of low load that qPCR failed to identify. The detailed information is shown in **Table 1**.

**Table 1:** The mNGS and qPCR results of all samples.

Name	Sample	qPCR	mNGS	Pathogen read number	RPM
Patient 1	BALF	Positive	Positive	20	20.75
	Blood	Negative	Negative	0	0
Patient 2	BALF	Positive	Positive	338	390.43
Patient 3	BALF	Negative	Positive	23	30.2
	Blood	Negative	Negative	0	0
Patient 4	BALF	Negative	Positive	4	3.88
	Blood	Negative	Positive	37	15.42
Patient 5	BALF	Positive	Positive	659	339.55
	Blood	Negative	Positive	3	0.79
Patient 6	BALF	Positive	Positive	3,293	9,327.1
Patient 7	BALF	–	Positive	10,127	557.16
Patient 8	BALF	Positive	Positive	235	21.93
Patient 9	BALF	–	Positive	375	17.71
Patient 10	BALF	Negative	Positive	7	0.49
Patient 11	BALF	–	Positive	8	0.53
Patient 12	BALF	Negative	Positive	871	131.08
	Blood	Negative	Positive	8	0.36
Patient 13	BALF	–	Positive	74	4.88
Patient 14	BALF	–	Positive	137	2137
Patient 15	BALF	Negative	Positive	14	0.83
Patient 16	BALF	–	Positive	98	29.81

The reads number and the reads per million (RPM) of detected Chlamydophila psittaci were calculated.

### The Comparison of BALF-mNGS and Blood-mNGS

**Figure 3:**
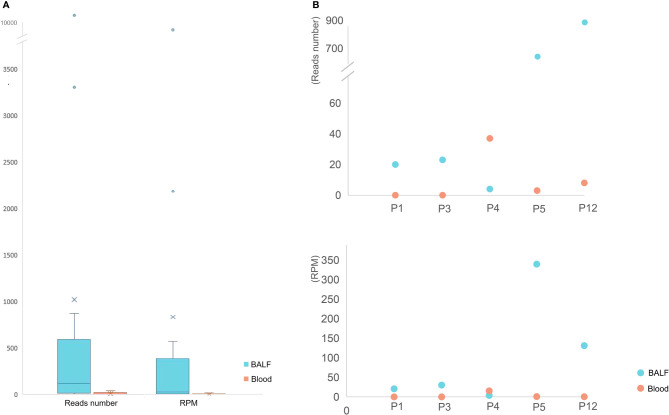
The mNGS results of BALF and blood samples. **(A)** The read number and RPM of *C. psittaci* in BALF samples were significantly higher than those of blood samples. **(B)** The read number (top) and RPM (bottom) of *C. psittaci* of both BALF and blood samples in five cases. P1 and P3 were negative by mNGS of blood.

BALF and blood samples are commonly used sample types for mNGS detection, and the mNGS results of different types of samples were compared among the 16 BALF and five blood samples collected (**Figures 3A, B**). The mNGS efficiency (the percentage of microbial reads) of BALF and blood samples were 10.27% and 9.25%, respectively. Various microbial reads were detected by both blood and BALF mNGS, indicating a similar efficiency of blood and BALF for mNGS detection. However, when only considering the pathogen of psittacosis, all BALF samples gave positive results, but blood samples in two out of five patients failed to detect the pathogen (**Figure 3B**). The overall positive rate of BALF-mNGS (100%) was significantly higher than that of blood mNGS (60%) (*p* < 0.05). In addition, the detected read number and its RPM of BALF-mNGS were also significantly higher than those of blood-mNGS (*p* < 0.01) (**Figure 3A**). This indicated that BALF is a more relevant sample type to detect the pathogen of psittacosis.

### The Comparison of mNGS With Normal or Larger Datasets

**Figure 4:**
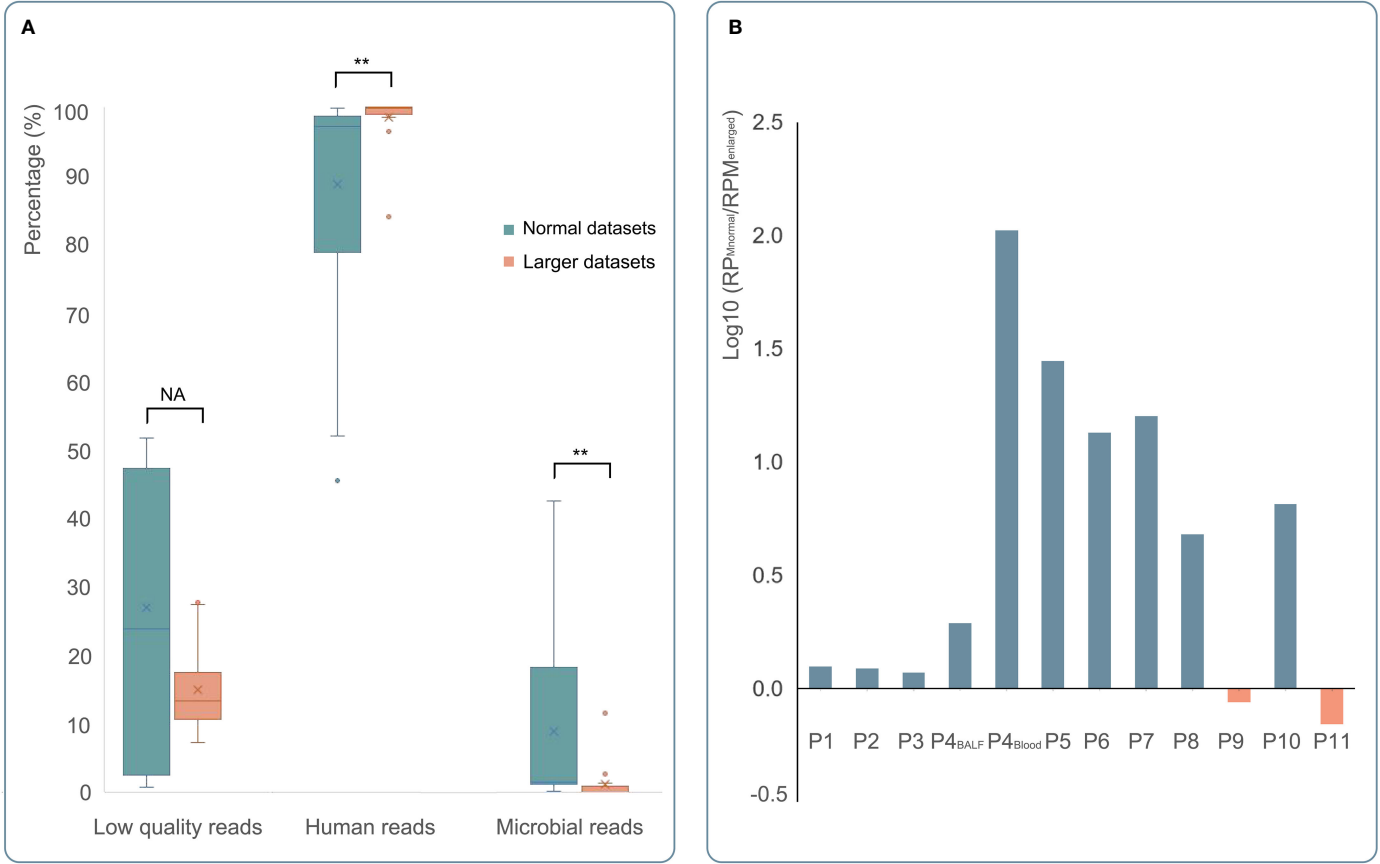
The comparison of mNGS detection results with normal or larger datasets. **(A)** The results of mNGS with normal datasets and larger datasets were shown in green and orange, respectively. After enlarging the datasets of mNGS, the content of human DNA increased significantly (*p < *0.01), and the mNGS efficiency (the percentage of microbial reads) decreased significantly (*p < *0.01). **(B)** Except three blood samples (one was positive by mNGS with normal datasets); the remaining detection results of 12 samples showed positive by mNGS with larger datasets. Compared to mNGS with normal datasets, a decreased RPM of *C. psittaci* was found in 10 samples (blue bar). Only two samples (orange bar) showed a little higher RPM by mNGS with larger datasets. *p < 0.05, **p < 0.01 , and NA p > 0.05.

mNGS with larger datasets was performed in 15 samples from 11 patients in this study. The read count has tripled, and the base number was 12-fold larger compared to clinical mNGS with a normal dataset. The percentage of low-quality reads, human host reads, and microbial reads and the specific read number and RPM of *C. psittaci* were calculated (**Figure 4A**). After comparing the clinical mNGS detection results with normal data volume, it was found that larger datasets generated a higher content of human DNA (98.46% ± 4.11 vs. 88.70% ± 17.76) and a lower mNGS efficiency (1.14% ± 2.97 vs. 8.94% ± 14.75) (*p* < 0.01), while there was no significant difference in low-quality read percentage before and after dataset adjustment. The detected read number and RPM of *C. psittaci* were further compared. The specific read number increased, but the RPM drastically decreased after increasing the datasets. Except three blood samples (one was negative after increasing the sequence data, and two were negative both before and after increasing the sequence data of mNGS), the remaining detection results revealed a decreased RPM of *C. psittaci* after increasing the sequence data in 10 samples (0.01~0.85 times). Only two samples (1.15 and 1.44 times) showed a slight increase in RPM after increasing the sequence data of mNGS (**Figure 4B**), indicating that the increase in the amount of mNGS sequencing data might not result in a better detection result.

### Whole-Genome Capture and Phylogenetic Tree

**Figure 5:**
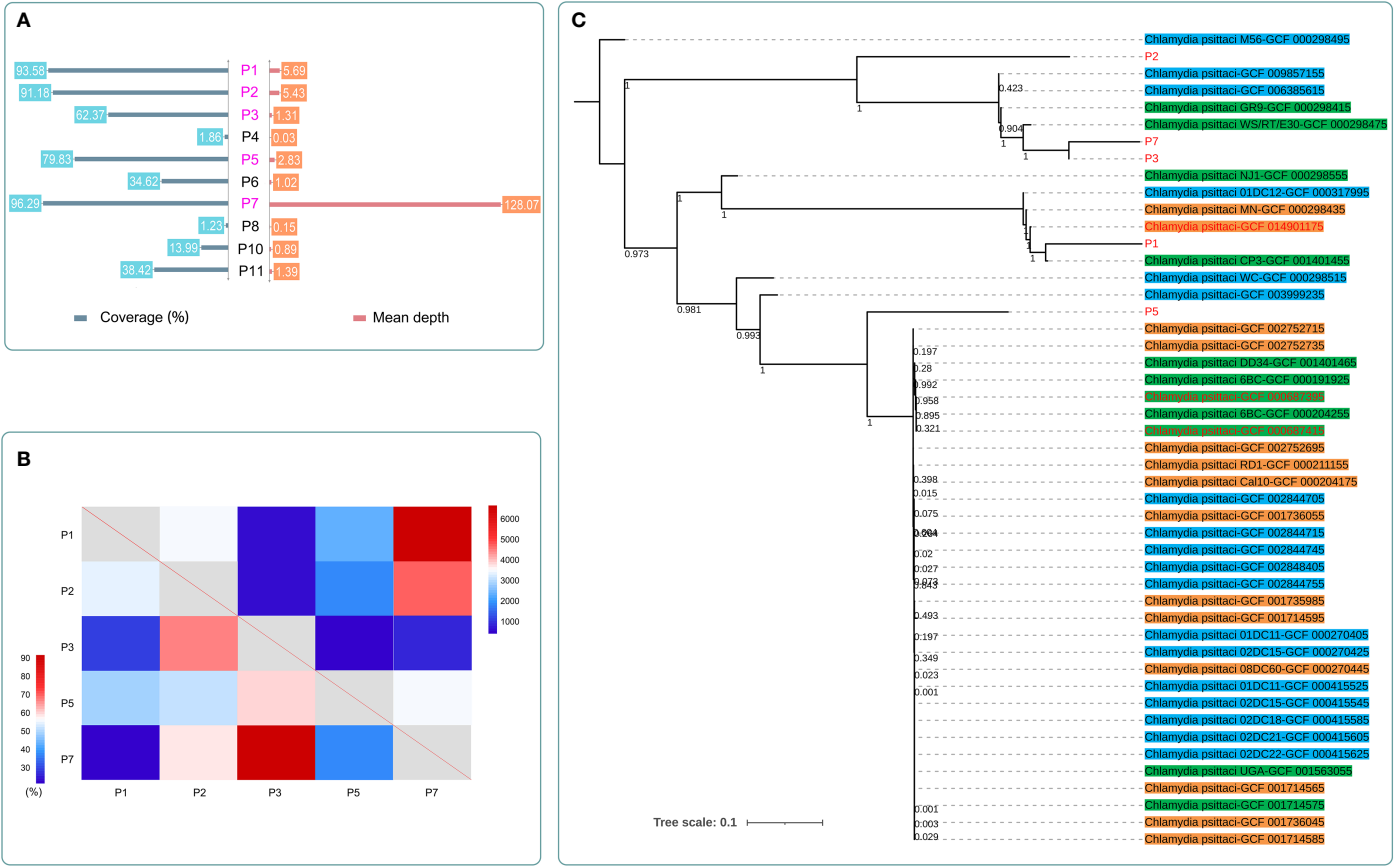
The detection results after whole-genome capture and the maximum-likelihood phylogenetic tree of *C. psittaci* in this study. **(A)** The coverage and mean depth of 10 samples after capture. The data of *C. psittaci* in five cases (marked in purple) with coverage >60% and mean depth >1 were further analyzed. **(B)** The detected SNP number (top right) and the shared variation percentage (bottom left) of each paired samples after capture. P3 and P7 showed the closest genetic relationship among the five samples; they shared the same variations in 92% of 856 SNPs. **(C)** The maximum-likelihood phylogenetic tree of *C*. *psittaci.* Samples marked in red were from China. Samples highlighted in green were isolated from birds. Samples highlighted in blue were isolated from mammals. Samples highlighted in orange were isolated from human.

Whole-genome capture of *C. psittaci* was performed in 10 patients (**Figure 5A**). The coverage ranged from 96.29% to 1.23%, and the mean depth ranges from 128.07 to 0.03. The number of detected SNPs and the shared variations of each paired sample after capture are shown in **Figure 5B**. There were 856 SNPs detected in both P3 and P7, and the variations of 92% of these SNPs were the same, indicating their closest genetic relationship than other paired isolates. Five samples with coverage >60% and mean depth >1 were finally used to construct the phylogenetic tree and were clustered into different clusters, with all branches of *C. psittaci* able to infect various hosts, including birds (parrots, pigeons, ducks, and chickens), mammals (horses, cattle, and pigs), and human (**Figure 5C** and **S1**). The five samples in this study and three other *C. psittaci* samples from China were clustered separately in multiple sub-branches with those samples from other regions worldwide. A high genetic diversity of *C. psittaci* in China and no obvious geographical distribution characters of *C. psittaci* were demonstrated. Only *C. psittaci* from P3 and P7 showed a very close relationship, confirming the SNP analysis (**Figure 5B**). Interestingly, P3 and P7 both had a history of contact with birds, and their closest *C. psittaci* sample (GCF_000298475) was also isolated from birds. P1 which was most closely related with GCF_001401455 isolated from a pigeon also had a history of exposure to pigeon. In addition, P1 also had a close relationship with GCF_014901175, which was isolated from an ICU patient with severe pneumonia from China.

## Discussion

Psittacosis caused by *C. psittaci* is a systemic infectious disease with a pulmonary component, of which the clinical diagnosis by conventional methods is always difficult and easy to be ignored (Gregory and Schaffner, 1997; Rybarczyk et al., 2020). When diagnosing psittacosis, qPCR has a relatively higher sensitivity and specificity than serology and culture, but it requires *a priori* hypothesis of target pathogens (Mitchell et al., 2009). However, *C. psittaci* is not always first considered by clinicians, and its symptoms are often atypical, which limit the use of qPCR in diagnosing psittacosis. Unlike qPCR, mNGS can unbiasedly detect almost all organisms in the sample without *a priori* hypothesis and has been widely used for the clinical diagnosis of various infections with satisfying performance. In this study, mNGS also performed a significantly higher sensitivity than qPCR (*p* < 0.01), indicating its greater potential in diagnosing psittacosis, especially when the DNA load of the pathogen is low. Thus, the pan detection spectrum and high sensitivity of mNGS allow the early and accurate diagnosis of diseases with atypical symptoms, which can help the adjustments of antibiotic treatment. In addition, BALF has been considered as a promising sample type for the diagnosis of psittacosis in this paper, while blood can still serve as an alternative for mNGS, especially for patients who cannot tolerate bronchoscopy examination.

mNGS can detect pathogens with an extremely low DNA load in the sample. In this study, five BALF samples and five blood samples were detected as negative by qPCR of *C. psittaci*, but mNGS succeeded to identify the pathogen in 8 of the 10 samples. The sensitivity of qPCR was relatively low. The reason may be that the primers of qPCR in this study are not optimal. They can only be used to detect limited genotypes of *OmpA*. In fact, we analyzed the *OmpA* region of each sample using the whole-genome capture data. Reads that are mapped to each *OmpA* genotype with identity ≥95 and coverage ≥95 were recorded (**Table S2**). Except three samples with no reads mapped to the *OmpA* region, seven samples successfully detected the genotypes. They can be clustered into genotypes A, B, E, and EB, all of which might be detected by our qPCR primers. qPCR was performed in five of the seven patients. The sensitivity of qPCR was 80% (4/5), while mNGS detected in all of the five samples. Further analysis to compare the value of mNGS and qPCR in diagnosing psittacosis is required.

Compared to blood samples, BALF performed better for the diagnosis of psittacosis. The reason could be that the DNA load of the pathogen in BALF may be higher than that in blood. As a semiquantitative tool (Zhang et al., 2020), mNGS revealed a higher DNA read number and RPM of the pathogen in BALF than in blood samples, confirming our hypothesis. It is known that BALF is a relevant sample type for the diagnosis of lung infections, but many patients could not tolerate bronchoscopy examination. In addition, this invasive procedure may bring in other pathogens, causing another infection, especially for immunocompromised patients (Jolis et al., 1996). Our findings also indicated that blood could be an alternative for the early diagnosis of psittacosis using mNGS, although the DNA load of pathogens was lower than BALF.

The low load of the pathogen in samples may be the main limitation for the early diagnosis. Therefore, we evaluated if the increased amount of mNGS sequencing data could improve the diagnosis. Theoretically, the sensitivity of mNGS is based on the amount of detected reads of the pathogen. The more reads are detected, the higher the sensitivity of mNGS is determined. Therefore, it would be assumed that a higher amount of sequencing data may improve the mNGS detection. However, it was shown that the content of human DNA increased. Although the read counts tripled, the percentage of microbial reads decreased significantly after enlarging the datasets of mNGS, indicating its lower mNGS efficiency. For the pathogenic microorganism, although the specific read number increased, the RPM drastically decreased. In fact, there was one blood sample, which was detected as positive with a normal dataset, which turned negative after a larger dataset of mNGS. Therefore, enlarging datasets of mNGS could not improve the mNGS detection and might even result in a worse result. A previous study by Arnt Ebinger *et al.* also confirmed our finding (Ebinger et al., 2021). They explored the threshold for the routine application of mNGS in the clinical diagnostic context and found that the impact of the dataset size was neglectable for mNGS detection. Our findings indicated that larger datasets of mNGS can carry even more host and background compositions than pathogen compositions and complicate the further analysis.

The epidemiological analysis of pathogens is always difficult. mNGS cannot cover enough genetic information due to their low contents in clinical samples. Culture is commonly used to clone and isolate the pathogen. However, there are many pathogens that are not able or hard to be cultivated, such as viruses. Target capture can enrich the pathogen’s DNA reads of the library from those of human and environment microbes prior to sequencing (Samorodnitsky et al., 2015); the coverage has significantly increased, for example, from 1.23% to 96.29% of P7 in this study, suggesting that capture may be a potential way for epidemiological analysis.

Psittacosis is a zoonotic disease; most patients have experienced a history of exposure to birds, especially parrots, pigeons, and poultry (Kaleta and Taday, 2003; Stewardson and Grayson, 2010). Therefore, this disease is always eliminated from diagnostic consideration with a negative contact history (Cunha, 2006). However, there were nearly half of the patients (43.75%) with psittacosis who stated no contact with birds in this study. In addition, the high genetic diversity of *C. psittaci* in China and its complex epidemiology also indicate that the impact of this disease in pneumonia patients may be underestimated. It should be noted that empirical antibiotic therapy, such as β-lactam antibiotics, is often given to patients with suspected bacterial pneumonia, but it may have little efficacy for psittacosis (Cunha, 2006). The delay of treatment may cause worse prognosis of patients and even death. Therefore, mNGS should be applied as quickly as possible in patients with suspected psittacosis. The early and accurate diagnosis can help the timely adjustment of antibiotics, improving the prognosis of patients.

In this study, we introduced mNGS to psittacosis patients, which showed an ideal performance for the early diagnosis of this disease. Although an increased amount of sequencing data for mNGS could not improve the diagnosis, the high sensitivity of mNGS with normal sequencing data indicated that blood could be an alternative for the early diagnosis of psittacosis using mNGS, especially when bronchoscopy examination is not feasible. Considering the high genetic diversity of *C. psittaci* in China and its complex epidemiology, the early diagnosis of psittacosis using mNGS is needed, which can help the timely adjustment of antibiotic treatments and improve the prognosis. However, the sample size of this study was limited. More samples are needed to further evaluate the value of mNGS using BALF and blood in the early diagnosis of psittacosis and other infectious diseases.

## Data Availability Statement

The datasets presented in this study can be found in online repositories. The names of the repository/repositories and accession number(s) can be found as follows: https://www.cncb.ac.cn/, PRJCA007480.

## Ethics Statement

This study was approved by the ethical review committee of the General Hospital of the People’s Liberation Army (No. S2019-266-02). Written informed consent to participate in this study was provided by the participants’ legal guardian/next of kin.

## Author Contributions

ZD and FX designed the paper. ZD, YG, ZL, and YZ drafted the manuscript. BL, BS, SL, CW, DL, and KW were involved in the clinical care and management of the patients. ZD, FX, and ZL revised the manuscript. All authors contributed to the article and approved the submitted version.

## Funding

This study is supported by National Natural Science Foundation of China (61976223), Military Program (BLB20J002), National Key Research Program of China (2019YFC0121703), Chinese PLA General Hospital Program (CX19030, 2017MBD-011, 2018FC-WJFWZX-2-04), and the Science and Technology Project of Xi'an (No. 21RGSF0013).

## Conflict of Interest

ZL and YZ are employed by Hugobiotech Co., Ltd. The reviewer XXM declared a shared parent affiliation with the authors YG, SL to the handling editor at the time of review.

The remaining authors declare that the research was conducted in the absence of any commercial or financial relationships that could be construed as a potential conflict of interest.

## Publisher’s Note

All claims expressed in this article are solely those of the authors and do not necessarily represent those of their affiliated organizations, or those of the publisher, the editors and the reviewers. Any product that may be evaluated in this article, or claim that may be made by its manufacturer, is not guaranteed or endorsed by the publisher.

## Supplementary Material

Supplementary Figure 1 The neighbor-joining phylogenetic tree of *C. Psittaci* in this study.
