# Supplementary figures and images for: The Application Value of Metagenomic and Whole-Genome Capture Next-Generation Sequencing in the Diagnosis and Epidemiological Analysis of Psittacosis
Source: Front Cell Infect Microbiol. 2022 Jun 6;12:872899. doi: 10.3389/fcimb.2022.872899 (PMC9207344; doi:10.3389/fcimb.2022.872899)

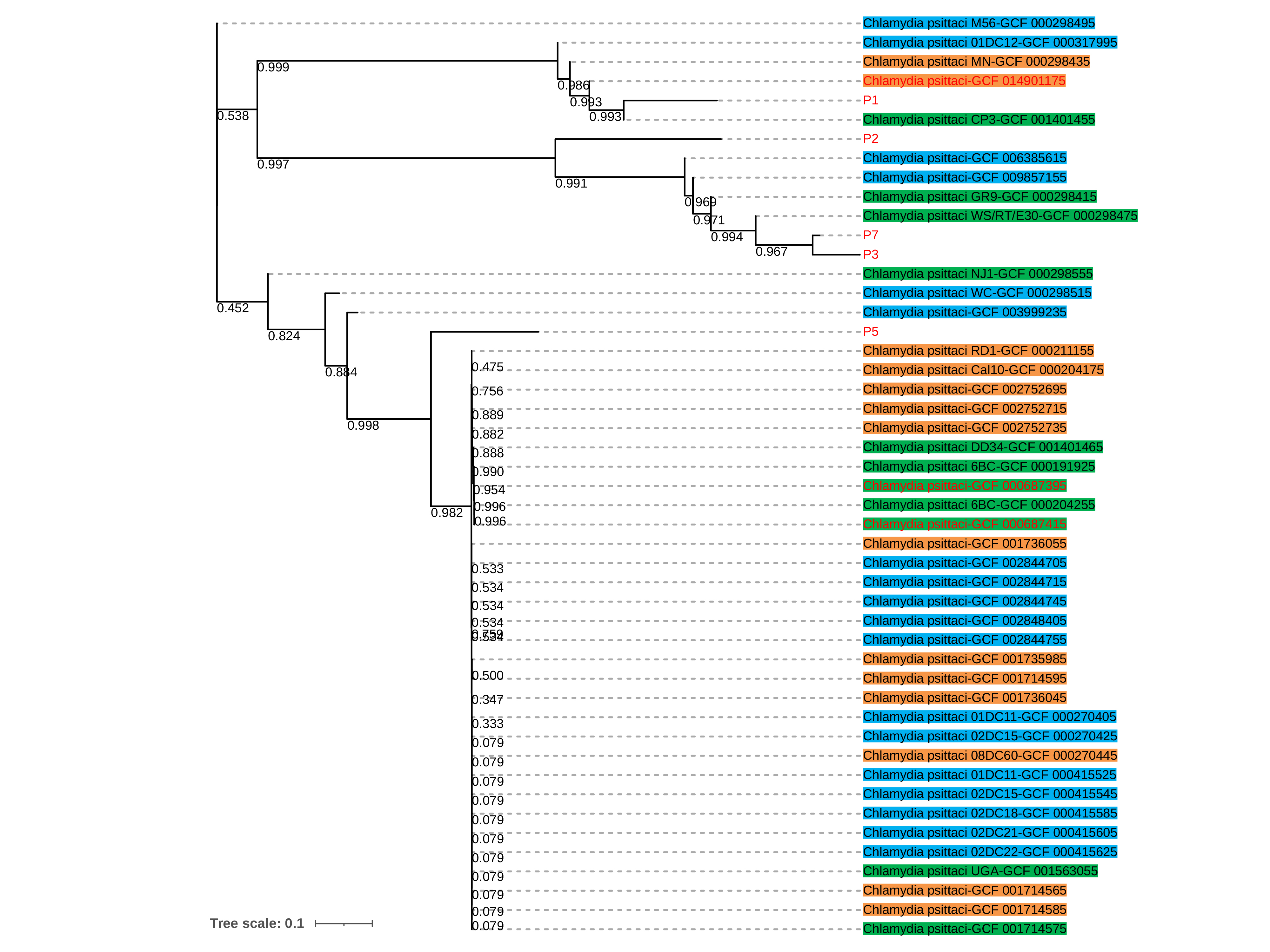

Supplement: Supplementary Figure 1 — The neighbor-joining phylogenetic tree of C. Psittaci in this study. [file Image_1.jpeg]
